# Identification of Crotonylation Metabolism Signature Predicting Overall Survival for Clear Cell Renal Cell Carcinoma

**DOI:** 10.1155/2023/5558034

**Published:** 2023-11-28

**Authors:** Jie Zheng, Yingqing Liu, Kai Wei, Jiewu Shi, Lin Li, Xuefeng Jiang, Lingsong Tao

**Affiliations:** ^1^Department of Urology, Wuhu Hospital Affiliated to East China Normal University, Wuhu 241000, Anhui, China; ^2^Department of Urology, The Second Affiliated Hospital of Soochow University, Suzhou, Jiangsu 215004, China

## Abstract

**Background:**

Immunotherapy shows promise in treating cancer by leveraging the immune system to combat cancer cells. However, the influence of crotonylation metabolism on the prognosis and tumor environment in ccRCC patients is not fully understood.

**Methods:**

We conducted various systematic analyses, including prognosis and cluster analyses, to investigate the role of KAT2A in immunotherapy. We used qRT-PCR to compare KAT2A expression in cancer and adjacent tissues and among different cell lines. Additionally, we employed Cell Counting Kit-8, wound healing, and Transwell chamber assays to assess changes in the proliferative and metastatic ability of A498 and 786-O cells.

**Results:**

We identified three clusters related to crotonylation metabolism, each with distinct prognosis and immune characteristics in ccRCC. We categorized CT1 as immune-inflamed, CT2 as immune-excluded, and CR3 as immune-desert. A new system, CRS, emerged as an effective predictor of patient outcomes with differing immune characteristics. Moreover, qRT-PCR revealed elevated KAT2A levels in ccRCC tissues and cell lines. KAT2A was found to promote ccRCC and correlate significantly with immunosuppressive elements and checkpoints. Reducing KAT2A expression hindered ccRCC cell growth and metastasis.

**Conclusion:**

Our study highlights the critical role of crotonylation metabolism in cancer development and progression, particularly its link to poor prognosis. CRS proves to be an accurate predictor of patient outcomes and immune features in ccRCC. KAT2A shows strong associations with clinical factors and the immunosuppressive environment, suggesting potential for innovative immunotherapies in ccRCC treatment.

## 1. Introduction

Post-translational modification (PTM) of proteins encompasses chemical alterations that occur after the translation process. These modifications wield substantial influence over intracellular signal transduction, metabolism, and gene regulatory networks by orchestrating changes in protein stability, activity, localization, and interactions with other biological macromolecules, such as proteins, nucleic acids, and lipids [1–3]. Consequently, they hold sway over a wide array of cellular functions. In normal cells, PTMs play a pivotal role in precisely and swiftly regulating cell proliferation, thereby dictating the cell's state—be it quiescence or proliferation. In contrast, in cancer cells, PTMs can foster abnormal proliferation by steering cell cycle-related effector proteins and perpetuating proliferation signals [4–6]. The vast spectrum of PTMs, numbering in the hundreds, encompasses forms like phosphorylation, glycosylation, acetylation, ubiquitination, and acylation [7]. Among them, protein acylation stands out as a pivotal post-translational modification. Lipidated proteins often share an intimate association with non-polar structures like lipid bilayers, greatly elevating their hydrophobicity and, in turn, modulating their conformation, membrane affinity, localization, and mobility [8]. Consequently, protein acylation plays a pivotal role in regulating cell proliferation and metabolism, regardless of whether the context is normal or cancerous.

Histone crotonylation, a conservative and non-acetylated histone lysine acylation modification, occupies a prominent niche in transcriptional regulation and disease progression [9, 10]. Lysine crotonylation (Kcr), a subtype of histone lysine acylation, primarily unfolds at the *ε*-amino group of histone lysines [11]. The mechanisms behind histone crotonylation's establishment, removal, and recognition are orchestrated by well-known enzymes involved in histone acetylation [12]. Furthermore, localized shifts in histone crotonylation levels can precipitate corresponding changes in gene expression [13]. Importantly, histone crotonylation exerts distinctive biological functions, impacting cell metabolism, the cell cycle, tissue development, and other vital processes [14–16]. Nonetheless, the prognostic implications and underlying biological mechanisms of Kcr in clear cell renal cell carcinoma (ccRCC) remain veiled.

This study embarks on a systematic exploration of the prognostic significance and immune signatures associated with Kcr-related genes in ccRCC. We also construct Kcr-related clusters distinguished by their varying prognostic and immune characteristics. Leveraging data from the Cancer Genome Atlas (TCGA) and the E-MATB-1980 dataset, we construct and validate a Kcr-based prognostic model to predict the fate of ccRCC patients. Additionally, we delve into the clinical attributes, biological pathways, and immune properties of KAT2A, a pivotal gene in Kcr modification.

## 2. Methods

### 2.1. Data Acquisition and Processing

Transcriptome data and clinical information of ccRCC patients were obtained from TCGA and Gene Expression Omnibus (GEO) databases. A set of 17 crotonylation-related genes (CRGs) were curated from pertinent literature sources [17, 18]. The GSE22541 dataset was utilized as external validation datasets, while the GSE36895 and GSE73731 datasets were utilized to evaluate the clinical features of the model genes.

### 2.2. Construction of Crotonylation-Related Clusters

To evaluate the role of CRGs in tumor progression, we adopted the non-negative matrix factorization (NMF) algorithm to classify patients. Survival differences between crotonylation-related clusters were evaluated using Kaplan–Meier (KM) analysis. To further delve into the differences of biological pathways among the clusters, we identified differentially expressed genes (DEGs) according to the criteria of | log-Fold Change (logFC) > 1.5 | and adjusted *p* value <0.01. We then conducted Gene Ontology (GO) and Kyoto Encyclopedia of Genes and Genomes (KEGG) enrichment analyses to unravel the underlying mechanisms of these DEGs. Gene set variation analysis (GSVA) was employed to explore variations in pathway enrichment among the clusters.

### 2.3. Establishment of the Crotonylation-Related Signature

Firstly, we performed the Wilcoxon test to discern the differential expression of 17 CRGs between cancer and adjacent tumors tissues. Subsequently, we subjected the differentially expressed CRGs to univariate Cox regression analysis to identify CRGs associated with prognosis. Utilizing the expression profile of prognostic related CRGs, we further constructed a prognostic model using the least absolute shrinkage and selector operation (LASSO) analysis. The crotonylation-related signature (CRS) was obtained by linear combination of gene expression weighted regression coefficients. The algorithm was as follows: CRS = Coef A *∗* Gene A expression + Coef B *∗* Gene B expression + Coef C *∗* Gene C expression+……Coef N *∗* Gene N expression, with Coef referring to the coefficient calculated by LASSO and gene expression referring to the expression of CRGs. According to the ratio of 1 : 1, the patients were classified into training and test group. The survival differences in overall survival (OS, overall survival is a measure of the length of time individuals or patients survive from a defined starting point (such as diagnosis or treatment initiation) until death from any cause) were analyzed between the high and low groups. The time-dependent receiver operating characteristic (ROC) curve and univariate and multivariate analyses were adopted to assess the stability and accuracy of the model. The GSE22541 dataset was used for external validation of the model.

### 2.4. Evaluation of the Immunogenomic Landscape

Multiple algorithms were exploited to investigate immune infiltration characteristics of ccRCC samples. The Spearman algorithm was employed to analyze the correlation between CRS and immunoinfiltrating cells. The anticancer immune response (cancer immune cycle) in the tumor microenvironment (TME) had seven steps. The immune activity scores on ccRCC samples were collected from the Tracking Tumor Immunophenotype (TIP, https://biocc.hrbmu.edu.cn/TIP/). Tumor microenvironment (TME) may affect the occurrence and development of cancer, so we employed the ESTIMATE algorithm to evaluate the TME score (ImmuneScore, StromalScore, and tumor purity) of ccRCC samples. Additionally, the single-sample gene set enrichment analysis (ssGSEA) algorithm was applied to assess immune function pathway scores in ccRCC samples.

### 2.5. Screening and Validation of Hub Crotonylation-Related Genes

On the basis of modeled genes expression, the recursive feature elimination- (RFE-) support vector machine (SVM) algorithm was utilized to further screen the hub crotonylation-related genes. Then, the KM survival curve and ROC curve were adopted to evaluate the prognostic characteristics of hub genes. We further exploited the correlation between hub genes and clinicopathological variables. Then, the real-time quantitative PCR (RT-qPCR) was utilized to evaluate the differential expression of modeling genes between ccRCC and normal tissues. Protein expression data for KAT2A between cancer and paracancer tissues were retrieved from the Human Protein Atlas (HPA) database.

### 2.6. Cell Culture and Cell Transfection

Two human ccRCC cell lines (A498 and 786-O) were purchased from the cell bank of the Chinese Academy of Sciences (Shanghai, China). All cells were cultured in RPMI 1640 medium (Thermo Fisher Scientific, Inc.) supplemented with 10% fetal bovine serum (FBS; Thermo Fisher Scientific, Inc.) at a constant temperature of 37°C in a humidified atmosphere containing 5% CO_2_.

Lentiviral shRNA plasmids that target KAT2A together with the nonspecific control shRNA were obtained from Dharmacon (Shanghai, China). Transfection of plasmid and shRNA was performed with Lipo3000 following the manufacturer's instructions.

### 2.7. Cell Counting Kit-8 (CCK8) Assay

In brief, A498 and 786-O cells after different interventions were incubated in 96-well plates (2 × 10^3^), supplemented with 200 *μ*L culture medium and conditioned in 37°C with 5% CO_2_. On days 1, 2, 3, 4 and 5, 20 *μ*L CCK8 solution was added into each well, and incubation was performed for 2 h. Absorbance was measured at an optical density of 450 nm using a microplate reader (Bio-Rad Laboratories, Inc.).

### 2.8. Transwell Assay

A498 and 786-O cells (with an cubation density of 2 × 10^5^) were incubated in the upper chambers (Corning). For the invasion assay, the upper chambers were precoated with Matrigel (BD Biosciences). Culture medium without and with 10% FBS was added into the upper and lower chambers, respectively. After 12 h, non-migrated cells were wiped out while migrated or invaded CRC cells were fixed, stained, and counted using an inverted microscope.

### 2.9. Wound Healing Assay

Cell migration was assessed by performing a wound healing assay. In brief, A498 and 786-O cells were transfected with KAT2A. Approximately 2 × 10^6^ cells were seeded into 6-well plates and cultured for 24 h. Then, a yellow plastic pipette tip was used to create a wound by scraping the cells. Cell migration was monitored under a Nikon Eclipse microscope and photographed at 100×.

## 3. Results

### 3.1. Establishment of the Crotonylation-Related Clusters and Functional Enrichment Analysis

**Figure 1:**
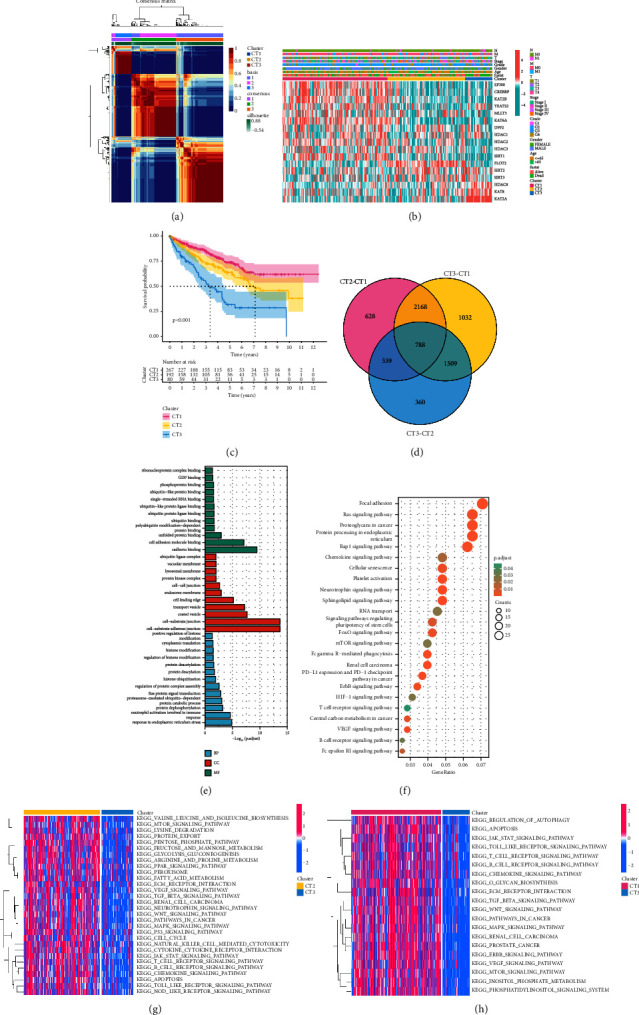
Construction of crotonylation metabolism-related clusters. (a) Different crotonylation metabolism-related molecular clusters of the TCGA cohort were identified for *k* = 3. (b) Differences in crotonylation metabolism-related gene expression and clinical characteristics among the clusters. (c) KM survival curve of crotonylation metabolism-related molecular clusters. (d) Common differential genes among clusters. (e) Representative enriched GO terms of differential genes among clusters. (f) Representative enriched KEGG terms of differential genes among clusters. (g, h) Representative enriched GSVA terms between clusters.

We utilized the NMF algorithm to establish three disparate crotonylation metabolism-related clusters (CT1, CT2, and CT3) according to 17 CRG expressions (Figure 1(a)). The heatmap illustrated the distribution of the clinical variables and CRG expression (Figure 1(b)). The KM curve indicated that CT1 patients had the best prognosis (Figure 1(c)). To delve into difference of pathway enrichment among the clusters, we screened the DRGs according to the criteria of | logFC| ≥ 1.5 and adjusted *p* value <0.01. Venn diagram demonstrated that 788 DEGs were common among the three distinct crotonylation metabolism-related clusters (Figure 1(d)). GO analysis revealed that DEGs were mainly enriched in cadherin binding, cell adhesion molecule binding, cell-substrate junction, and protein catabolic process (Figure 1(e)). KEGG analysis suggested that DEGs were focused on the immune-related pathway, oncogenic pathway, and angiogenesis pathway (Figure 1(f)). Additionally, GSVA analysis unveiled that CT1 was significantly related to the enrichment pathway related to immune activation, matrix and carcinogenic activation pathway were significantly enriched in CT2, and CT3 was obviously associated with the biological process of immunosuppression (Figures 1(g) and 1(h)).

### 3.2. Immune Infiltration Characteristics of Crotonylation-Related Clusters

**Figure 2:**
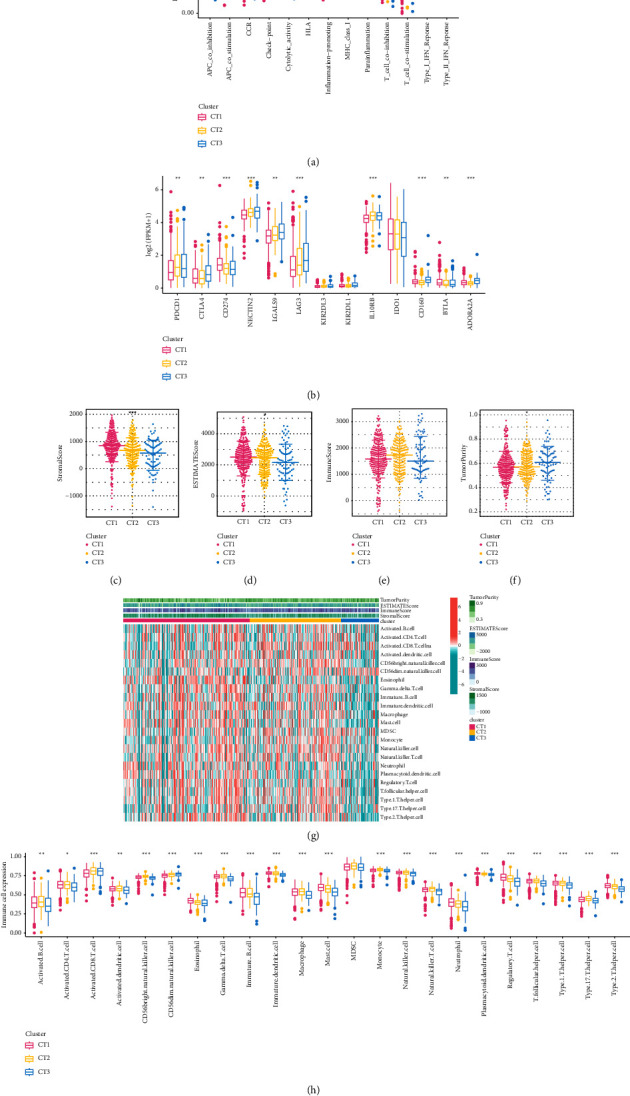
Immune infiltration characteristics of crotonylation metabolism-related clusters. (a) Differential expression of immune function score among different crotonylation metabolism-related clusters. (b) Differences in immunosuppressive checkpoint expression among crotonylation metabolism clusters. (c–f) Differences in tumor microenvironment scores among crotonylation metabolism-related clusters. (g) Distribution of immune cell and tumor microenvironment scores among crotonylation metabolism clusters. (h) Differences in immune inflating cells among crotonylation metabolism-related clusters.

To further explore causes of survival differences among crotonylation metabolism-related clusters, we analyzed characteristics of immune infiltration. Initially, we analyzed differences of immune function pathways among the clusters, finding that the expression of multiple immune function pathways was highest in CT1 and lowest in CT3 (Figure 2(a)). Immunosuppression checkpoints were differentially expressed among crotonylation metabolism-related clusters, and the highest expression was found in CT3 (Figure 2(b)). The TME scores showed significant differences among various groups, with CT1 exhibiting the highest expression in StromalScore and ESTIMATEScore, while displaying the lowest expression in tumor purity (Figures 2(c)–2(f)). Heatmap presented the distribution of immune infiltrating cells and TME scores among crotonylation metabolism-related clusters (Figure 2(g)). Additionally, the expression of immune infiltrating cells was the highest in CT1 and the lowest in CT3 (Figure 2(h)).

### 3.3. Establishment of the Crotonylation-Related Signature and Prognosis Analysis

**Figure 3:**
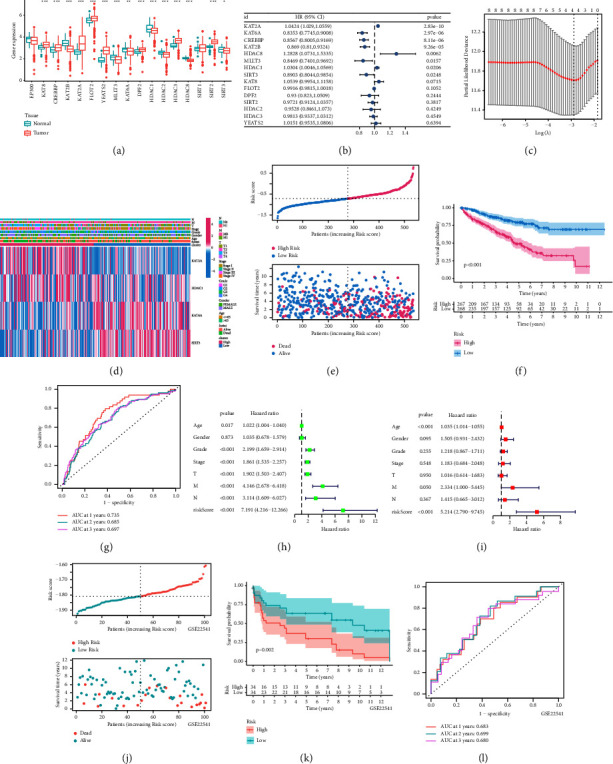
Construction and validation of the crotonylation metabolism-related signature. (a) Differences in crotonylation metabolism-related genes expression between ccRCC and normal tissues. (b) Results of univariate Cox regression analysis. (c) LASSO regression identified 4 crotonylation metabolism-related genes. (d) Distribution of modeled genes and clinicopathologic features between crotonylation metabolism signatures. (e) The risk curve of each sample reordered by crotonylation metabolism-related signature and the distribution of survival states. (f) Survival analysis of the crotonylation metabolism signature. (g) ROC curves about crotonylation metabolism-related signature in 1–3 years. (h, i) The results of univariate and multivariate Cox analysis of crotonylation metabolism-related signature. (j) Risk curve of each sample reordered by crotonylation metabolism signature and the distribution of survival states in GSE22541. (k) Survival analysis of the crotonylation metabolism signature in GSE22541. (l) ROC curves about crotonylation metabolism signature in 1–3 years in GSE22541.

To explore the role of CRGs in ccRCC, we explored differential expression of CRGs between cancer and paracancerous tissues. Except for EP300 and SIRT1, the expression of other 15 CRGs was significantly different between ccRCC and paracancer tissues (Figure 3(a)). Subsequently, we identified eight prognostic related CRGs through univariate Cox regression analysis (Figure 3(b)). After performing LASSO regression analysis on these 8 genes, we selected 4 genes to establish the crotonylation-related signature (Figure 3(c)). Patients were randomly divided into training group and test group on a 1 : 1 ratio. Heatmap illustrated the distribution of four modeled genes and clinical variables in allrisk group (Figure 3(d)). KM curve and survival status distribution suggested that patients in the high CRS group had a worse prognosis in the allrisk group (Figures 3(e) and 3(f)). ROC curves about CRS in one, two, and tree years in the allrisk group were 0.735, 0.685, and 0.697 (Figure 3(g)). Univariate and multivariate Cox analysis suggested that CRS had strong predictive accuracy for the prognosis of ccRCC patients (Figures 3(h) and 3(i)). GSE22541 served as an external validation dataset to verify stability of CRS. The KM curve and survival status distribution in the GSE22541 showed that CRS was associated with poor prognosis (Figures 3(j) and 3(k)). ROC curves about CRS in 1, 2, and 3 years in the GSE22541 dataset were 0.683, 0.699, and 0.680 (Figure 3(l)). Similar results were obtained in the training and test risk groups (Figure S1).

### 3.4. Identification of Clinicopathological and Prognostic Characteristics of the Crotonylation-Related Signature

**Figure 4:**
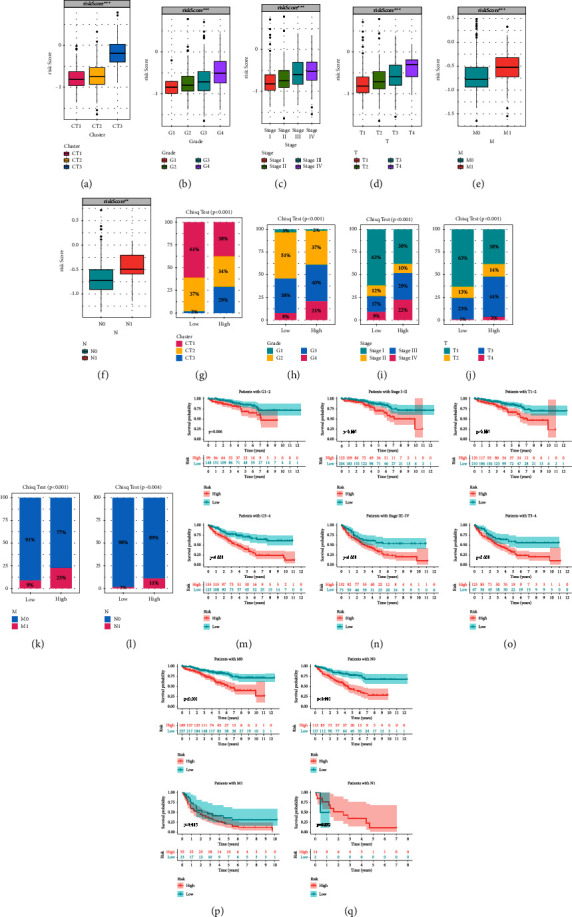
Correlation analysis between crotonylation metabolism signature and clinicopathological stages. (a–f) Differences in crotonylation metabolism signature among clinicopathological variables. (g–l) The histogram showing the proportion of clinicopathological variables in crotonylation metabolism signature groups. (m–q) Survival analysis of palmitoylation metabolism-related signature in different clinicopathological variables.

We assessed the correlation between CRS and clinicopathological variables by examining the differential expression of CRS in different clinical variables. CRS expression was highest in CT3, and its expression was more pronounced in advanced clinicopathologic variables (Figures 4(a)–4(f)). The chisq test was then utilized to analyze the differences in the distribution of clinicopathological variables between high and low CRS. Figures 4(g)–4(l) reveal that compared with low CRS group, the proportion of advanced clinicopathologic variables in high CRS group was higher and there was statistical difference. The KM curves indicated that among different clinicopathological variables, the high CRS group was associated with poor prognosis and had significant differences (Figures 4(m)–4(q)). These results corroborated the association between higher CRS expression and worse patient prognosis.

### 3.5. Identification of Immune Infiltration Characteristics of the Crotonylation-Related Signature

**Figure 5:**
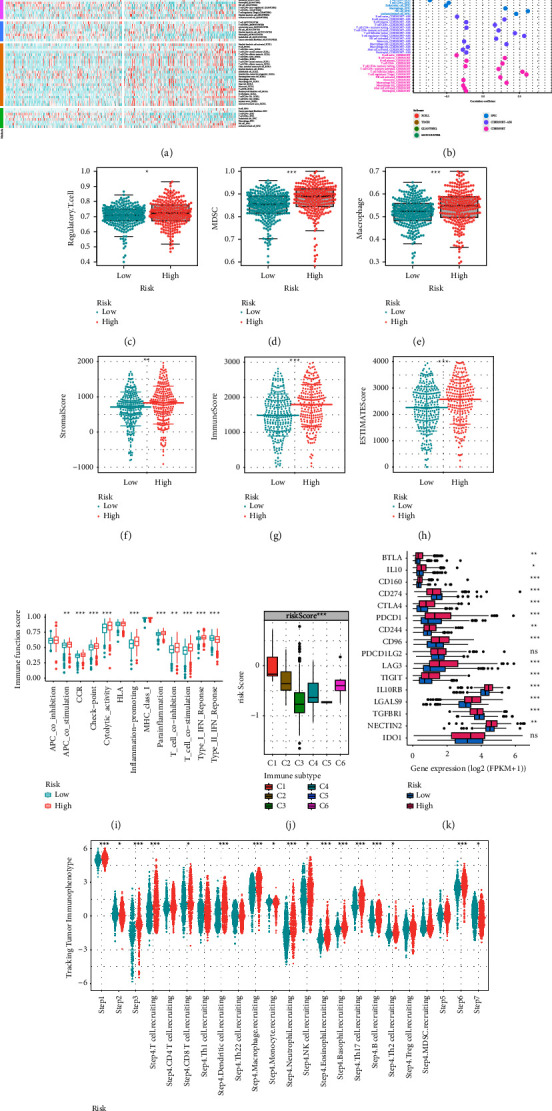
Identification of the immune characteristics of the crotonylation metabolism signature. (a) Heatmap representing expression of immune cells in crotonylation metabolism-related signature groups under various algorithms. (b) Correlation analysis of immune cells and crotonylation metabolism signature under multiple algorithms. (c–e) Expression difference of immunosuppressive cells in crotonylation metabolism-related signature groups. (f–h) Expression difference of tumor microenvironment scores in crotonylation metabolism-related signature groups. (i) Difference of immune function scores in crotonylation metabolism-related signature groups. (j) Differential expression of crotonylation metabolism signature among immune subtypes. (k) Expression difference of immunosuppressive checkpoints in crotonylation metabolism-related signature groups. (l) Differential expression of crotonylation metabolism signature in tracking tumor immunophenotypes.

We further evaluated the correlation between CRS and immunoinfiltrating cells. Across various algorithms, the expression distribution of immune cells in the high and low-risk groups were analyzed (Figure 5(a)). Additionally, we analyzed correlation coefficient between CRS and immune cells by Spearman algorithm and visualized it in the lollipop chart (Figure 5(b)). Immunosuppressive cells (Regulatory T cell, Myeloid-Derived Suppressor Cells (MDSCs), and Macrophage) were obviously overexpressed in high CRS group (Figures 5(c)–5(e)). Besides, the StromalScore, ImmuneScore, and ESTIMATEScore were all significantly overexpressed in the high CRS group (Figures 5(f)–5(h)). The high CRS group exhibited higher expression levels in immune functional pathways such as the checkpoint, cytoolytic activity, CCR, and inflammation-promoting pathway (Figure 5(i)). In order to further assess the relevance of CRS to immune typing, we then analyzed the relation between CRS and previously reported pan-cancer immune subtypes. The expression of CRS was higher in C1, C2, and C6 and lowest in C3 (Figure 5(j)). Given that C6 was correlated with a poor prognosis and C3 was linked to a better prognosis, these results suggested a unique characteristic of the ccRCC immune microenvironment. Most immunosuppressive checkpoints were significantly overexpressed in the high CRS group (Figure 5(k)). Antitumor immunity must effectively eliminate cancer cells through a gradual process. To further analyze function of immune cells in progression of ccRCC, we obtained immune activity score of each step in ccRCC sample from TIP. Antitumor immune cells were significantly overexpressed in high CRS group (Figure 5(l)).

### 3.6. Identification of Prognostic Characteristics of Hub Crotonylation-Related Genes

**Figure 6:**
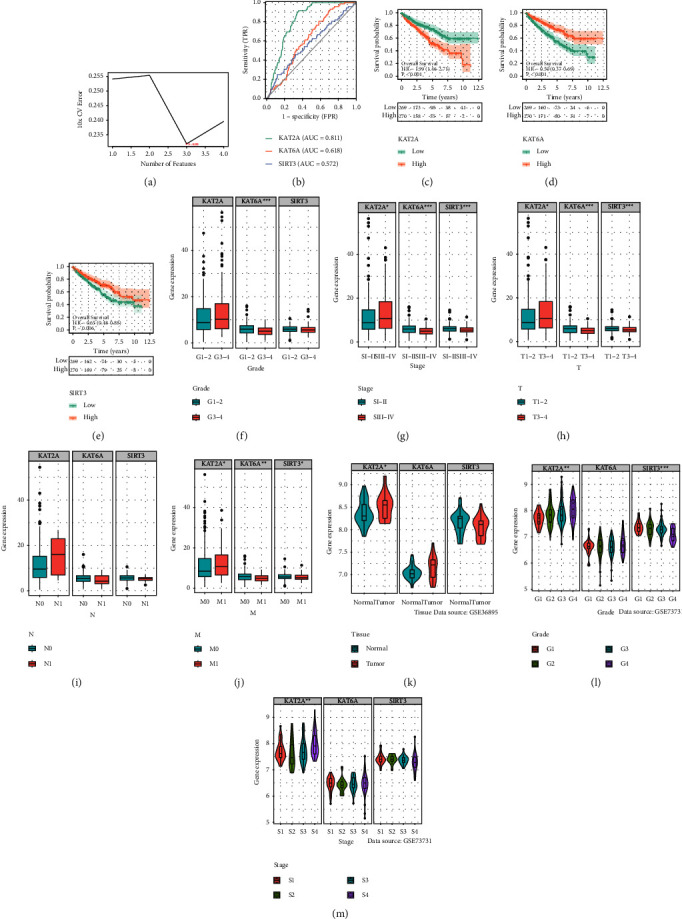
Clinicopathological characteristics and prognostic characteristics of hub crotonylation metabolism-related genes. (a) Optimal outcome of screening ccRCC genes by SVM algorithm. (b) ROC curves of three hub crotonylation metabolism-related genes. (c–e) Survival analysis of the three hub crotonylation metabolism-related genes. (f–m) Expression difference of three hub crotonylation metabolism-related genes in various clinicopathological stages in TCGA and GEO datasets.

In order to identify the most representative prognostic genes related to crotonylation modification in RCC, we employed the SVM-RFE method to screen three genes (KAT2A, KAT6A, and SIRT3) (Figure 6(a)). The AUC of ROC was used for evaluation of the ability to predict the prognosis of patients with ccRCC on the basis of gene expression. The AUC values of KAT2A, KAT6A, and SIRT3 were 0.811, 0.618, and 0.572, respectively (Figure 6(b)). KM analysis showed that KAT2A was associated with a poor prognosis, while KAT6A and SIRT3 displayed an inverse association (Figures 6(c)–6(e)). Furthermore, KAT2A expression was higher in advanced clinical variables, while KAT6A and SIRT3 were opposite (Figures 6(f)–6(m)).

### 3.7. Identification of Immune Characteristics of KAT2A

**Figure 7:**
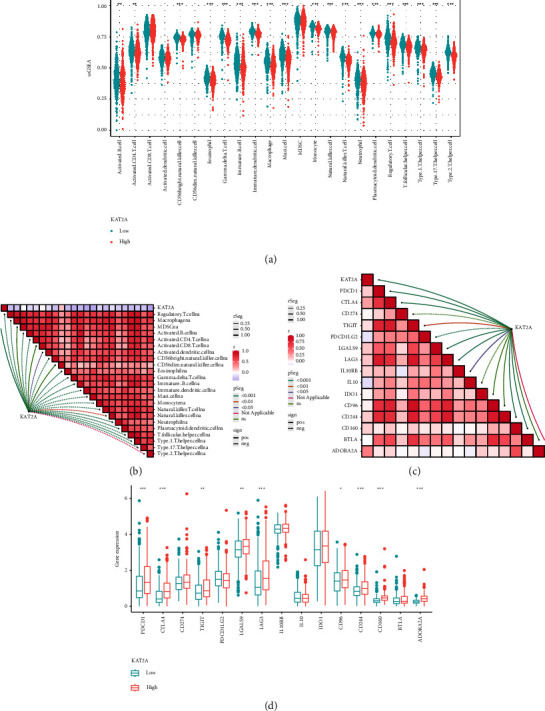
Immunological characteristics and expression verification of KAT2A. (a) Expression difference of immune cells in KAT2A groups. (b) Correlation analysis between immune cells and KAT2A. (c) Correlation analysis between immunosuppression checkpoints and KAT2A. (d) Expression difference of immunosuppression checkpoints in KAT2A groups.

Then, we further analyzed immune characteristics of KAT2A. Patients were grouped into high and low KAT2A expression groups based on the average expression of KAT2A. In the low KAT2A group, most immune cells were markedly upregulated (Figure 7(a)).  Figure 7(b) shows a significant negative correlation between KAT2A and a variety of immune cells. Moreover, KAT2A was positively correlated with multiple immunosuppressive checkpoints, with most of these checkpoints being markedly overexpressed in the high KAT2A group (Figures 7(c) and 7(d)).

### 3.8. KAT2A Knockdown Suppressed Proliferation, Migration, and Invasion in A498 and 786-O Cells

**Figure 8:**
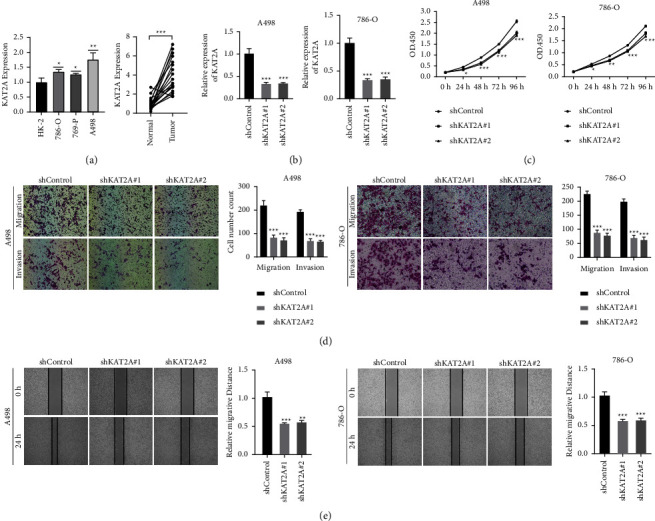
Downregulation of KAT2A suppressed the progression of ccRCC in vitro. (a) mRNA differential expression of KAT2A in ccRCC tissues and cell lines. (b) The expression of KAT2A in A498 and 786-O cells was detected by RT-qPCR. (c) KAT2A knockdown suppressed ccRCC cell proliferation in A498 and 786-O cells. (d) KAT2A knockdown suppressed ccRCC cell metastasis in A498 and 786-O cells. (e) Wound healing tests demonstrated changes in ccRCC cell migration.

In addition, 18 pairs of ccRCC tissues, tree ccRCC cell lines, and 1 normal renal cell line were detected by RT-qPCR. KAT2A was markedly upregulated in tumor tissues. Furthermore, KAT2A was markedly upregulated in ccRCC cell lines and was highest in A498 cell line in comparison to normal kidney cell lines (Figure 8(a)). In the KAT2A knockdown group, mRNA and protein expression of KAT2A were dramatically downregulated (Figure 8(b)). The CCK8 assay demonstrated that the proliferation of A498 and 786-O cells was markedly decreased in KAT2A knockdown group (Figure 8(c)). Transwell experiments revealed that the migration and invasion of A498 and 786-O cells were clearly inhibited in KAT2A knockdown group (Figure 8(d)). Wound healing detection suggested that the healing distance of A498 and 786-O cells in KAT2A knockdown group was lower than that in control group after 24 hours (Figure 8(e)). These findings indicated that the expression of KAT2A was positively correlated with the proliferation, migration, and invasion of ccRCC cells.

## 4. Discussion

Renal cell carcinoma (RCC) is an extremely complex tumor originating from epithelial cells, of which ccRCC is the most common subtype [19, 20]. The incidence and mortality of ccRCC were increasing year by year, accounting for about two to tree percent of adult malignancies [21–23]. Since ccRCC was insensitive to targeted and immunosuppressive agents, surgical treatment remains the main and most effective treatment [23, 24]. Despite significant progress in early screening and diagnosis, about one-third of patients already metastasized when diagnosed and about 25% have metastases after surgical treatment [25, 26]. Lysine crotonylation takes part in many biological processes, including translation initiation, RNA splicing, DNA damage and repair, cell cycle, and amino acid metabolism. This study focuses on understanding the potential role of crotonylation modification in ccRCC, as Kcr has been implicated in various cellular processes, including those related to cancer.

Immunotherapy has emerged as a promising approach for the treatment of cancer, offering several advantages over traditional therapies such as chemotherapy and radiation [27]. Its design is centered on the specific targeting of cancer cells while preserving healthy cells. Unlike conventional treatments that often harm both cancerous and healthy cells, immunotherapy is designed to target specific molecules or cells involved in the immune response against cancer. This targeted approach minimizes off-target toxicity and reduces the risk of side effects. Immunotherapy can reactivate the immune system, which can be particularly effective when cancer cells develop resistance to traditional treatments. Immune checkpoint inhibitors, for example, have shown success in blocking interactions that hinder immune responses, allowing immune cells to attack cancer cells more effectively.

In this study, based on the 17 CRG expressions, we employed the NMF algorithm to construct three crotonylation-modified clusters with different prognostic and immune characteristics. By analyzing the differences of immune and biological pathways between the three clusters, three crotonoylation modification clusters have markedly different TME cell infiltration characteristics. We speculated that a large number of immune cell infiltration and immune-related pathways were enriched in CT1, which was considered to be an immune-inflammatory type; a large number of innate immune cell infiltration and cancer-promoting activation-related pathways were enriched in CT2, and CT2 was considered an immune excluded type; there was a lack of immune cell infiltration in CT3, which was considered an immune desert type. However, CT2 was significantly enriched in innate immune cells but had a poorer prognosis. It has been shown that immune-excluded tumors were infiltrated by immune cells, but these immune cells were only present in the stroma surrounding the tumor cells. Therefore, activation of the stroma in the tumor microenvironment was considered to be T cell suppression [28]. In addition, matrix activation pathways were clearly enriched in CT2. These pathways include ECM receptor interaction, TGF-*β* signal pathway, and cell adhesion. Thus, we hypothesized that the antitumor effects of immune cells were suppressed by the activation of intermediates in the CT2 cluster. Subsequently, the univariate cox and Lasso analyses were utilized to structure the Crotonacylation modification-related prognosis model and verified the stability of this model in predicting patient prognosis with univariate and multivariate independent prognostic analysis and E-MATB-1980 datasets. In TME, invasive immune cells had key function in tumor proliferation, migration, invasion, and regulation of anticancer immunity and were extremely important therapeutic targets [29]. In high CRS group, tumor microenvironment score and proportion of immunosuppressive cell infiltration were higher, and the prognosis was worse. In conclusion, the crotonylation modification-related prognosis model was an important indicator for evaluating patients' prognosis and immune response, which was helpful for the formulation and development of personalized therapy for patients with ccRCC.

Kcr is not only a plentiful, evolutionally conservative, and physiologically related PTM but also significantly associated with the occurrence and progression of tumors. A quantitative proteomics study indicated that Kcr substrates targeted by P300 may be linked to cancer [30]. Besides, crotonylation modification was expressed differently in various cancers, such as high expression in thyroid, esophagus, colon, pancreas, and lung carcinomas, but low expression in liver and stomach carcinomas [31]. At the same time, in hepatocellular carcinoma (HCC), Kcr was correlated to tumor, lymph node, and metastasis (TMN) staging [14]. Besides histone crotonylation, many non-histone proteins also participate in carcinogenesis. In lung adenocarcinoma, many non-histone proteins were modified by crotonylation, and these proteins were significantly enriched in subcellular localization, cell composition, molecular function, and many important cellular pathways [32].

As the first writers, KAT2A (General control non-depressible 5 (GCN5)) has been shown to have acetyltransferase, succinyltransferase, and crotonyltransferase activities on histones [32–34]. KAT2A regulated multiple biological events and played a vital role in tumor initiation and progression [34]. KAT2A was highly expressed in non-small-cell radiation-induced lung cancer and may promote tumor progression by upregulating E2F1 and cyclin d1 [35]. GCN5 was overexpressed in HCC, and downregulation of KAT2A inhibits HCC cell and xenograft tumor proliferation [36]. KAT2A is markedly overexpressed in urothelial carcinoma, and KAT2A knockdown can inhibit the progression of urothelial carcinoma [37]. Our study revealed that KAT2A was markedly upregulated in ccRCC, and this conclusion was further verified by RT-qPCR assay and HPA dataset. At the same time, KAT2A was negatively associated with most immune cells and significantly positively correlated with immunosuppression checkpoints. We speculated that KAT2A might promote tumor metastasis and proliferation by participating in the establishment of an immunosuppressive tumor microenvironment.

Despite the valuable insights gained from this study, it has some limitations. These include the use of traditional univariate and Lasso regression analyses to construct the crotonylation-related prognosis model, which may benefit from more advanced methodologies. Additionally, the study's reliance on clinical information from the TCGA database, which may lack comprehensive data, could be complemented with additional parameters such as imaging data. Future research could focus on further elucidating the mechanisms through which crotonylation modification affects ccRCC progression and exploring the potential of targeted therapies, including immunotherapies, based on these findings. Moreover, clinical studies could be conducted to validate the prognostic and predictive value of the crotonylation-related signature in ccRCC patients, ultimately leading to more personalized treatment approaches for this challenging cancer subtype.

## 5. Conclusion

In brief, we classified ccRCC patients into three crotonylation metabolism-related clusters with different prognosis and immune cell infiltration characteristics. Moreover, the crotonylation metabolism-related prognostic model was constructed in ccRCC patients, which may be a marker to predict the prognosis and immune response of ccRCC patients. Meanwhile, KAT2A may contribute to the construction of an immunosuppressive tumor microenvironment, which may become a target for immunotherapy to further guide clinical treatment decisions.

## Data Availability

The data used to support the findings of this study are openly available in TCGA-KIRC and GEO (GSE22541, GSE36895, and GSE73731) datasets.

## Supplementary Materials

Supplementary Materials Figure S1: intra-group verification of crotonylation metabolism signature. (A, G) Distribution of modeled genes and clinicopathologic features in training and test groups. (B, H) The risk curve of each sample reordered by crotonylation metabolism-related signature and the distribution of survival states in training and test groups. (C, I) Survival analysis of the crotonylation metabolism signature in training and test groups. (D, J) ROC curves about crotonylation metabolism signature in 1, 2, and 3 years in training and test groups. (E, K) The univariate Cox analysis in training and test groups. (F, L) The multivariate Cox analysis in training and test groups.
