# Supplementary figures and images for: Identification of Crotonylation Metabolism Signature Predicting Overall Survival for Clear Cell Renal Cell Carcinoma
Source: Int J Clin Pract. 2023 Nov 28;2023:5558034. doi: 10.1155/2023/5558034 (PMC10697778; doi:10.1155/2023/5558034)

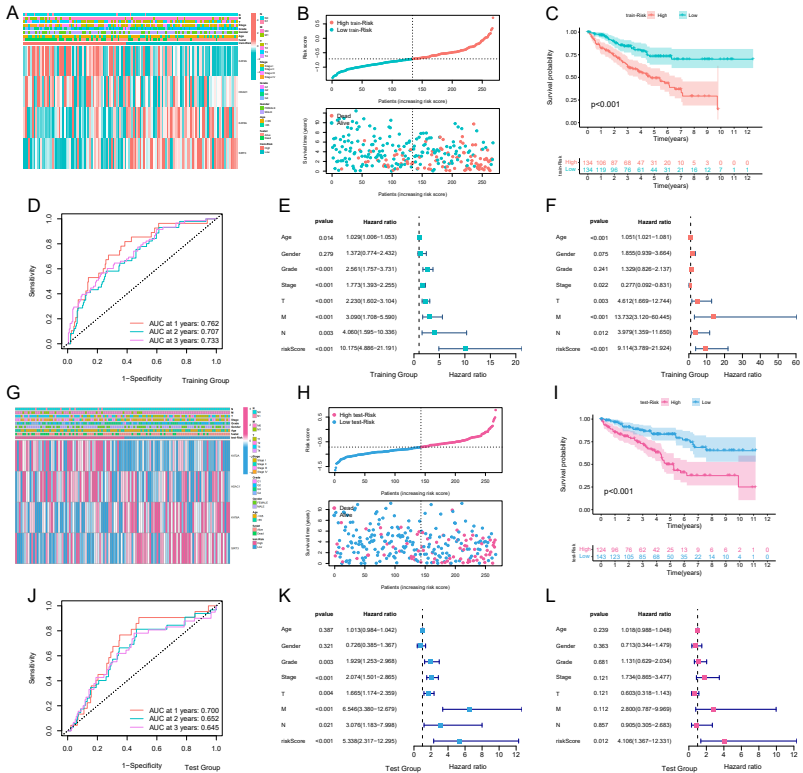

Supplement: Supplementary Materials — Figure S1: intra-group verification of crotonylation metabolism signature. (A, G) Distribution of modeled genes and clinicopathologic features in training and test groups. (B, H) The risk curve of each sample reordered by crotonylation metabolism-related signature and the distribution of survival states in training and test groups. (C, I) Survival analysis of the crotonylation metabolism signature in training and test groups. (D, J) ROC curves about crotonylation metabolism signature in 1, 2, and 3 years in training and test groups. (E, K) The univariate Cox analysis in training and test groups. (F, L) The multivariate Cox analysis in training and test groups. [file 5558034.f1.zip › Figure S1 (1).pdf]
